# Anaplastic lymphoma kinase overexpression enhances aggressive phenotypic characteristics of endometrial carcinoma

**DOI:** 10.1186/s12885-023-11144-2

**Published:** 2023-08-17

**Authors:** Ako Yokoi, Yusaku Nakamura, Miki Hashimura, Yasuko Oguri, Toshihide Matsumoto, Mayu Nakagawa, Yu Ishibashi, Takashi Ito, Kensuke Ohhigata, Youhei Harada, Naomi Fukagawa, Makoto Saegusa

**Affiliations:** 1 Department of Pathology, Kitasato University School of Medicine, 1-15-1 Kitasato, Minami-Ku, Sagamihara, Kanagawa 252-0374 Japan; 2 Department of Pathology, Kitasato University School of Allied Health Science, 1-15-1 Kitasato, Minami-Ku, Sagamihara, Kanagawa 252-0374 Japan

**Keywords:** ALK, Cancer stem cell, Epithelial-mesenchymal transition, Neuroendocrine differentiation, Endometrial carcinoma

## Abstract

**Background:**

Although anaplastic lymphoma kinase (ALK) is overexpressed in several primary solid tumor types, its role in endometrial carcinoma (Em Ca) remains unclear.

**Methods:**

We evaluated expression of ALK and its related molecules in clinical samples consisting of 168 Em Ca tissues. We also used Em Ca cell lines to evaluate the functional role of ALK.

**Results:**

Cytoplasmic ALK immunoreactivity in the absence of chromosomal rearrangement was positively correlated with ALK mRNA expression, and was significantly higher in Grade (G) 3 Em Ca than in G1 or G2 tumors. ALK immunoreactivity was also significantly associated with expression of cancer stem cell (CSC)-related molecules (cytoplasmic CD133, ALDH1, Sox2) and neuroendocrine markers (CD56 and synaptophysin). Although the proliferative index was significantly higher in ALK-positive Em Ca when compared to ALK- negative malignancies, there was no association between ALK expression and other clinicopathological factors in this disease. In Em Ca cell lines, full-length ALK overexpression increased proliferation, decreased susceptibility to apoptosis, enhanced cancer stem cell features, and accelerated cell mobility, whereas these phenotypes were abrogated in ALK-knockdown cells. Finally, patients with tumors harboring either wild-type *ALK* or high ALK mRNA expression had a poorer prognosis than those with either mutant *ALK* or low ALK mRNA expression.

**Conclusion:**

Full-length ALK overexpression occurs in a subset of Em Ca, particularly in G3 tumors, and contributes to the establishment and maintenance of aggressive phenotypic characteristics through modulation of several biological processes.

**Supplementary Information:**

The online version contains supplementary material available at 10.1186/s12885-023-11144-2.

## Introduction

The incidence of endometrial carcinoma (Em Ca), the most prevalent malignancy of the female genital tract in developing countries, is increasing [1, 2]. Although most frequently observed in post-menopausal women, 20—25% of Em Ca are diagnosed before the menopause [1, 2]. In Japan, the age-adjusted prevalence of Em Ca for women in 2014 was 16 per 100,000 and the overall rate has increased four-fold in the past 30 years, with a particularly rapid increase in women under 40 years old [3, 4]. The most common risk factors associated with the development of Em Ca are unopposed estrogen exposure and obesity (type I tumors); a smaller subset of sporadic Em Ca is associated with aging and unique genetic and molecular changes that produce a more aggressive variant (type II tumors) [5, 6]. Although most Em Ca patients are diagnosed at an early stage, 15—20% of cases present with advanced or recurrent disease and are associated with a 5-year survival rate of 17% [7, 8]. Thus, novel biomarkers and therapeutic targets for diagnosis or treatment of Em Ca are urgently required.

The *anaplastic lymphoma kinase (ALK)* gene located on chromosome 2p23 belongs to the insulin receptor superfamily of receptor tyrosine kinases (RTK), and encodes a protein that is highly homologous to leukocyte tyrosine kinase (LTK). ALK consists of a large extracellular domain, a lipophilic transmembrane segment, and a cytoplasmic tyrosine kinase domain [9–13]. During normal embryogenesis, ALK is specifically expressed in the developing central and peripheral nervous system, where it may regulate the balance between proliferation and differentiation [14–16]. However, this kinase also plays a role in disease pathology. For example, chromosomal rearrangements in a subset of lymphomas and lung carcinomas give rise to several gene fusions that contain ALK [10, 17]. Deregulated expression of full-length ALK has also been observed in some primary solid tumors including ovarian high-grade serous carcinoma, uterine carcinosarcoma, neuroblastoma, glioblastoma, and melanoma [18–22]. Thus, aberrant ALK overexpression is closely associated with tumor development and progression of multiple human malignancies.

Here, we provide clear evidence that overexpression of full length ALK in the absence of chromosomal rearrangements occurs in a subset of Em Ca. Using both ALK overexpression and knockdown, we also demonstrate a role for ALK in the modulation of proliferation, apoptosis, migration, and induction of cancer stem cell (CSC) features in Em Ca cell lines and primary tumor samples.

## Methods

### Clinical cases

Histological findings were reviewed in hysterectomy specimens of endometrioid-type Em Ca from the case records of Kitasato University Hospital between 2007 and 2020, according to the criteria of the 2014 World Health Organization classification [23]. Each case was also staged according to the 2009 International Federation of Gynecology and Obstetrics (FIGO) staging system and the TNM classification [24, 25]. A total of 168 Em Ca cases, including 91 of grade (G)1, 40 of G2, and 37 of G3 were investigated. The mean age of the patients was 59.1 years (range, 31—92), and 113 were post-menopausal. Detailed clinicopathological data are provided in Supplementary Table S1. All tissues were routinely fixed in 10% formalin and processed for embedding in paraffin wax. Approval for this study was given by the Ethics Committee of the Kitasato University School of Medicine (B18-048).

### Antibodies and reagents

Anti-ALK, anti-Rb phospho-Ser807/811 (pRb), anti-CD56, anti-synaptophysin (Syn), and anti-vimentin, anti-Slug, anti-cleaved caspase-3, and anti-cleaved poly (ADP-ribose) polymerase 1 (PARP1) antibodies were purchased from Cell Signaling (Danvers, MA, USA). Anti-Sox7, anti-ZEB1, and anti-β-actin antibodies were obtained from Sigma-Aldrich Chemicals (St. Louis, MO, USA). Anti-Snail, anti-Nestin, and anti-Sox2 antibodies were from Abcam (Cambridge, MA, USA). Anti-Rb, anti-p27^kip1^, anti-N-cadherin, anti-BAX, anti-X-linked inhibitor of apoptosis (XIAP), and anti-aldehyde dehydrogenase (ALDH)1 antibodies were from BD Biosciences (San Jose, CA, USA). Anti-Ki-67, anti-p21^waf1^, anti-cyclin D1, anti-p53, anti-BCL2, and anti-CD44s antibodies were from Dako (Glostrup, Denmark). Anti-cyclin A2, anti-cyclin B1, and anti-CD133 antibodies were from Novocastra (Newcastle, UK), Santa Cruz Biotechnology (Santa Cruz, CA, USA), and Miltenyi Biotechnology (Bergish Gladbach, Germany), respectively. Adriamycin (ADR: Catalog No. #D1515) was purchased from Sigma-Aldrich Chemicals.

### Immunohistochemistry (IHC)

IHC was performed using a combination of the microwave oven heating and polymer immunocomplex (Envision, Dako) methods. For immunohistochemical detection of ALK, the ALK iAEP kit (Nichirei Biosciences, Tokyo, Japan) was applied. Lung carcinoma tissues with ALK overexpression due to a gene abnormality were used as positive controls, as described previously [18, 19].

For evaluation of IHC findings, scoring of cytoplasmic immunoreactivity was performed on the basis of the percentage of immunopositive cells and the immunointensity with multiplication of the values of the two parameters as described previously [18, 19]. Samples with ALK score with more than 1 were considered positive and those with scores less than 1 were considered negative on the basis of the average ALK score (means ± SDs = 0.69 ± 1.65). Nuclear Ki-67 immunoreactivity was also counted in at least 500 cells from five randomly selected fields and the labeling indices (LIs) were then calculated as a percentage. In addition, the number of cleaved PARP1-positive cells in five randomly selected fields was used to calculate the mean number of apoptotic cells per high-power field (HPF), as described previously [18, 19].

### RNAscope assay for ALK mRNA in situ hybridization

Expression of ALK mRNA was analyzed using an RNAscope assay (Advanced Cell Diagnostics, Hayward, CA, USA) according to the manufacturer’s instructions. The hybridization was performed with targeted probes: Hs-ALK (#311841), positive control probe (#2010684), and negative control probe (#310043) for 2 h at 40℃. ISH signal scores were classified into four levels, as follows: -, none; 1 + , fewer than 10% positive cells; 2 + , 10–30%; 3 + , more than 30%.

### Plasmids and cell lines

pcDNA3.1-full-length ALK and pSIREN-RetroQ-short hairpin (sh) ALK were used as described previously [18, 19].

Eleven Em Ca cell lines (Ishikawa, Hec6, Hec50, Hec59, Hec88, Hec108, Hec116, Hec151, Hec155, Hec180, and Hec251), which we have established previously [26, 27], were used. Full-length ALK expression plasmid or empty vector were transfected into Hec6 cells (which lack endogenous ALK expression) (Supplementary Figure S1A) and two stably overexpressing clones (H6-ALK#8 and H6-ALK#47) were established. ALK-knockdown lines (ALK-KD) were also generated using Hec59 cells (which have relatively high ALK expression) (Supplementary Figure S1B) and shRNA targeting the *ALK* gene (H59-shALK#11 and H59-shALK#33) as described previously [18].

### Transfection

Transfection was carried out using LipofectAMINE PLUS (Invitrogen) as described previously [18, 19].

### Reverse transcription (RT)-PCR

cDNA was synthesized from 2 μg of total RNA. Amplification by RT-PCR was carried out in the exponential phase to allow comparisons between cDNA synthesized from identical reactions. Primers for the *ALK* and *GAPDH* genes were used as described previously [18, 19].

Western blot assays.

Total cellular proteins were isolated using RIPA buffer [20 mM Tris–HCl (pH 7.2), 1% Nonidet P-40, 0.5% sodium deoxycholate, 0.1% sodium dodecyl sulfate]. Aliquots of the proteins were resolved by SDS-PAGE and were transferred to PVDF membranes. After cutting the membrane on the basis of the protein sizes, they were probed with primary antibodies coupled to the ECL detection system (Amersham Pharmacia Biotechnology, Tokyo, Japan), as described previously [18, 19].

### Flow cytometry and aldefluor assay

Cells were fixed using 70% alcohol and stained with propidium iodide (Sigma) for cell cycle analysis. ALDH1 enzyme activity in viable cells was determined using a fluorogenic dye-based Aldefluor assay (Stem Cell Technologies, Grenoble, France) according to the manufacturer’s instructions. The prepared cells were analyzed by flow cytometry using BD FACS Calibur (BD Biosciences) and CellQuest Pro software version 3.3 (BD Biosciences), as described previously [18, 19].

### Spheroid assay

Cells (× 10^3^) were plated in low cell binding plates (Thermo Fisher Scientific, Yokohama, Japan) in Cancer Stem Cell Premium (ProMab Biotech, Richmond, CA). Uniform spheroids of at least 50 μm in diameter were counted approximately two weeks after plating, as described previously [18, 19].

### Wound healing assay

Cells were seeded into 24-well tissue culture plates, and grown to reach 90–100% confluence. After a cell monolayer formed, a wound was scratched with a sterile 200-μl tip. The area of the wound was also analyzed using ImageJ software version 1.41. Closure of the wound was measured in pixels, and used as a measure of cell migration, as described previously [18, 19].

### FIuorescence in situ hybridization (FISH)

For analysis of the *ALK* (2p23) locus, dual-color FISH studies were conducted on four HGSC cases with strong ALK immunopositivity using the Vysis LSI ALK break-apart rearrangement probe (Abbott Molecular, Abbott Park, IL, USA) according to the manufacturer’s instructions, as described previously [18, 19].

### Mutation analyses of the ALK gene

Genomic DNA was extracted from Hec59 cells using a Wizard Genomic DNA Purification kit (Promega, Madison, WT, USA) according to the manufacturer’s instructions. Mutation analyses of exons 20, 23, 24, and 25 of the *ALK* gene were carried out as described previously [18, 28]. Briefly, the PCR products were purified using a NucleoSpin Gel and PCR Clean-up (Macherey–Nagel, Düren, Germany) and bidirectional sequencing was performed using the BigDye Terminator v1.1 Cycle Sequencing Kit (Applied Biosystems, Foster City, USA) on the ABI3130 genetic analyzer (Applied Biosystems). Sequencing Analysis software v5.4 (Applied Biosystems) along with a manual chromatogram review was used for sequence analysis.

### Immunofluorescence

Hec6 cells stably overexpressing full-length ALK were incubated with anti-ALK antibody and Alexa 488 secondary antibodies (Thermo Fisher Scientific, Waltham, MA, USA) under the conditons described previously [19].

### TCGA data analysis

cBioportal (http://www.cbioportal.org/) was used to extract The Cancer Genome Atlas (TCGA) HGSA ALK mutational data and expression data (RNA Seq V2 PSEM) associated with 506 Em Ca cases. ALK mRNA expression levels were subcategorized into ‘high’ and ‘low’ groups on the basis of the Z score (cutoff values were 0.5). Cancer Cell Line Encyclopedia (CCLE) data describing the *ALK* gene status and the relative expression of ALK mRNA in 28 Em Ca cell lines were also extracted from cBioPortal, as described previously [18, 19].

### Statistics

Comparative data were analyzed using the Mann–Whitney *U*-test、chi-square rest, and Spearman’s correlation coefficient. Overall survival (OS) was calculated as the time between onset and death or the date of the last follow-up evaluation. Progression-free survival (PFS) was also examined from the onset of treatment until relapse, disease progression, or last follow-up evaluation. OS and PFS were estimated using the Kaplan–Meier method, and statistical comparisons were made using the log rank test. The cut-off for statistical significance was set as *p* < 0.05, as described previously [18, 19].

## Results

### ALK is overexpressed in a subset of Em Ca cases

**Fig. 1:**
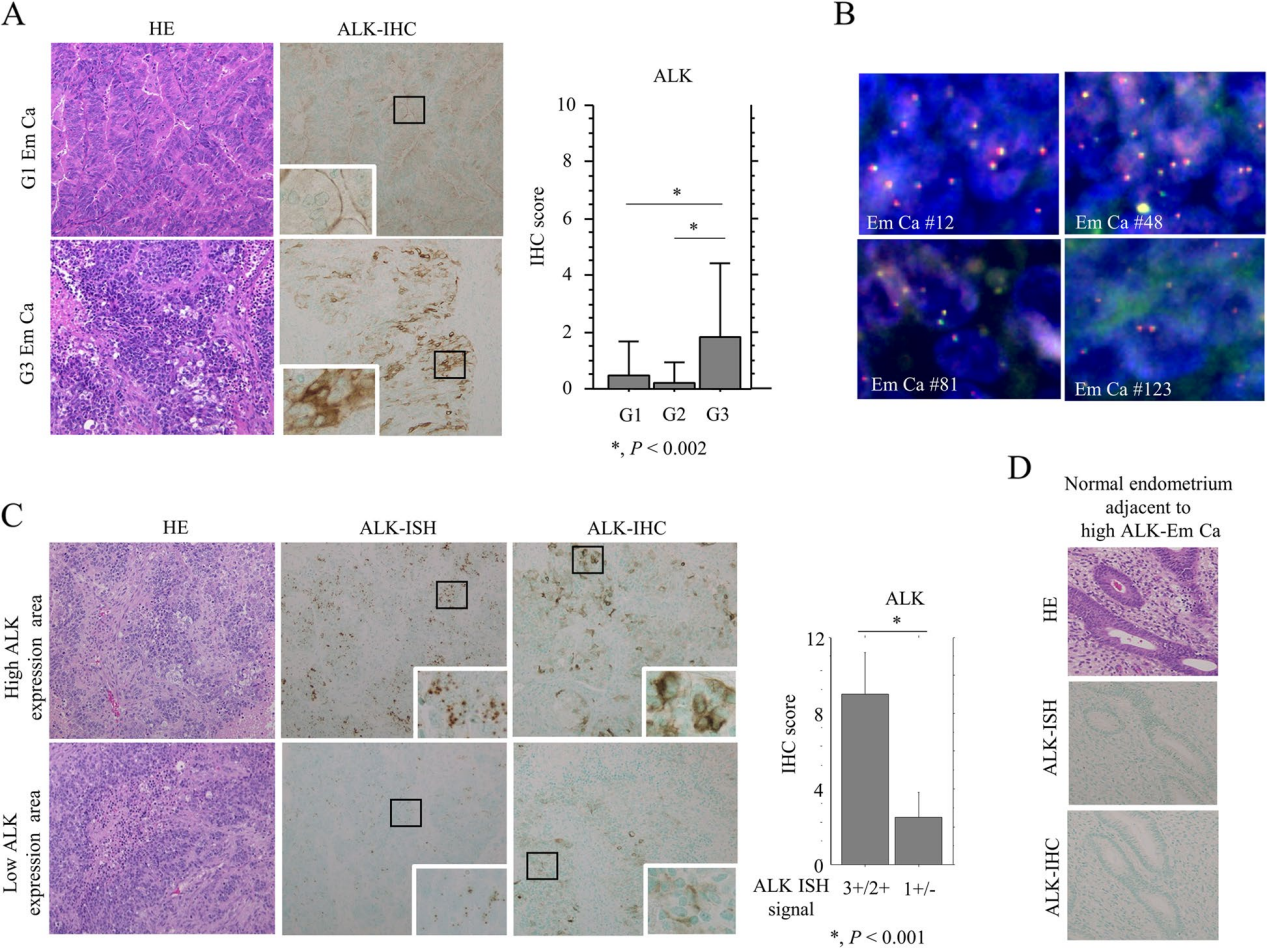
ALK mRNA and protein overexpression without gene alterations in Em Ca tissues. **A** Left: HE and IHC staining for ALK in G1 and G3 Em Ca. Note the strong cytoplasmic ALK immunoreactivity in G3 Em Ca, in contrast to the membranous immunoreactivity in G1 tumors. Closed boxes are magnified in the insets. Original magnification, × 100 and × 400 (inset). Right: IHC scores for ALK in Em Ca. The data shown are means ± SDs. Statistical analyses were carried out using the Mann–Whitney *U*-test. **B** FISH analysis of four Em Ca cases with high ALK scores. The interphase nuclei of both cases confirm the absence of *ALK* rearrangement, in which the red and green signals remain fused. Original magnification, × 400. **C** Left: HE, RNAscope (ISH), and IHC for ALK. Note the overlapping between ALK mRNA and protein expression in Em Ca tissues. Closed boxes are magnified in the insets. Original magnification, × 100 and × 400 (inset). Right: relationship between IHC scores and ISH signals for ALK in Em Ca. the data shown are means ± SDs. Statistical analyses were carried out using the Mann–Whitney *U*-test. **D** HE, RNAscope (ISH), and IHC for ALK in a case of normal endometrium adjacent to high ALK-immunopositive Em Ca. Note the lack of ALK mRNA and protein expression in endometrial glandular components. Original magnification, × 200

Representative images of IHC findings for ALK in Em Ca are illustrated in Fig. 1A. Strong cytoplasmic ALK immunoreactivity was mainly observed in G3 Em Ca, in contrast to the weak membranous immunoreaction in G1 tumors. ALK immunopositivity was observed in 14 (15.4%) of 91 G1 Em Ca, 3 (7.5%) of 40 G2, and 17 (45.9%) of 37 G3. Within the ALK-positive G1, G2, and G3 cases, cytoplasmic and membranous ALK immunoreactivities were observed in 7 (50%) and 7 (50%), 0 (0%) and 3 (100%), and 13 (76.5%) and 4 (23.5%) cases, respectively; the difference between G3 scores and the other tumor grades was statistically significant (Supplementary Table S1). A similar finding was also observed with regard to average ALK scores (Fig. 1A), whereas there were no associations between ALK expression and other clinicopathological factors in Em Ca (Supplementary Table S1). FISH did not reveal *ALK* rearrangement or amplification in four Em Ca cases with strong ALK immunoreactivity (Fig. 1B). ALK immunoreactivity clearly overlapped with ALK mRNA expression, and the ALK IHC scores were significantly higher in the ALK ISH-high category when compared to the ALK ISH-low group (Fig. 1C). In contrast, ALK was not expressed at the mRNA and protein level in normal endometrial components adjacent to the Em Ca tissues (Fig. 1D).

Among eleven Em Ca cell lines, two (Hec59 and Hec251) had relatively high ALK mRNA expression (Supplementary Figure S1A), in line with CCLE data analyses (Supplementary Figure S1B). However, ALK protein was only detected in Hec59 cells (Supplementary Figure S1C). Neither Hec59 nor Hec251 cells had mutations in exons 20, 23, 23, 24, and 25 of the *ALK* gene (Supplementary Figure S1D); these data are consistent with CCLE data that show there is no association between *ALK* gene mutation and levels of ALK mRNA (Supplementary Table S2).

Together, these findings suggest that cytoplasmic ALK expression without chromosomal rearrangement is observed together with increased ALK mRNA expression in G3 Em Ca.

ALK overexpression increases cell proliferation and decreases susceptibility to apoptosis in Em Ca cells.

To determine whether ALK played a functional role in Em Ca cells, we first established two independent Hec6 cell line clones stably overexpressing full-length ALK showing cytoplasmic, but not membranous, ALK localization (Supplementary Figure S1E), and two independent Hec59 cell line clones in which ALK expression was blocked by an ALK-specific shRNA.

**Fig. 2:**
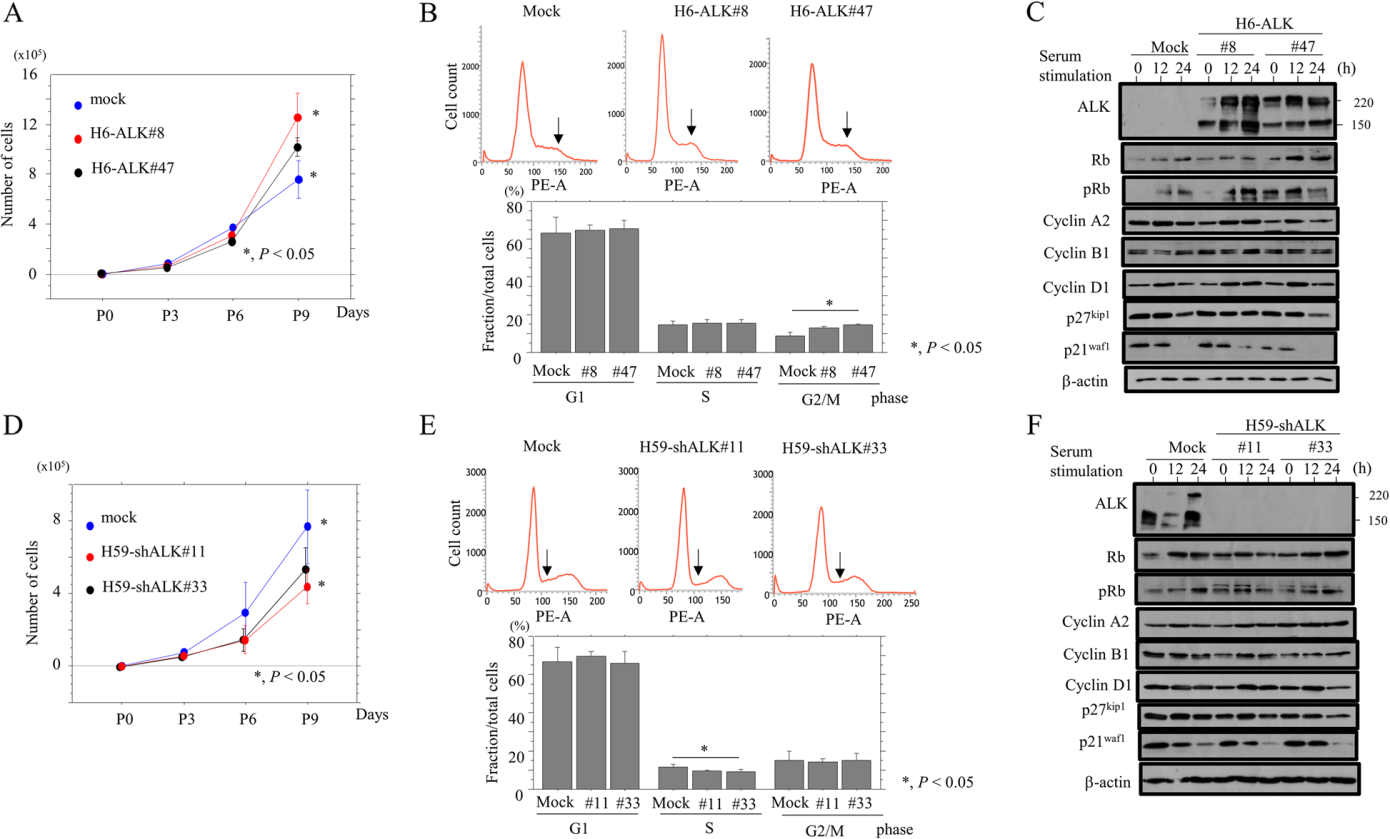
Relationship between ALK expression and proliferation in Em Ca cells. **A**, **D** Two independent H6-ALK (**A**), H59-shALK (**D**), and mock cells were seeded at low density. Cell numbers are presented as means ± SDs. P0, P3, P6, and P9 are 0, 3, 6, and 9 days after seeding, respectively. The experiments were performed in triplicate. Statistical analyses were carried out using the Mann–Whitney *U*-test. **B**, **E** Upper: Flow cytometry analysis of H6-ALK (B: G2/M phase is indicated by arrows), H59-shALK (**D**: S phase is indicated by arrows), and mock cells. Lower: percentage of G1, S, and G2/M fractions in H6-ALK (B), H59-shALK (**E**), and mock cells. The experiments were performed in quintuplicate. The data shown are means ± SDs. Statistical analyses were carried out using the Mann–Whitney *U*-test. **C**, **F** Right: western blot analysis for the indicated proteins in total lysates from H6-ALK (**C**), H59-shALK (**F**), and mock cells

To examine whether there was an association between ALK expression and cell cycle progression, we carried growth and serum starvation assays in H6-ALK and H59-shALK cells. H6-ALK cells tended to proliferate more rapidly than mock-transfected cells (Fig. 2A). This was consistent with the display of cell cycle progression markers including those associated with an increased G2/M fraction (Fig. 2B), increased Rb and pRb expression, and decreased p21^waf1^ expression (Fig. 2C and Supplementary Figure S2A). In contrast, H59-shALK cells tended to proliferate more slowly (Fig. 2D), and exhibited a reduced S phase fraction (Fig. 2E), although the expression of several cell cycle-related markers remained unchanged (Fig. 2F and Supplementary Figure S2B).

**Fig. 3:**
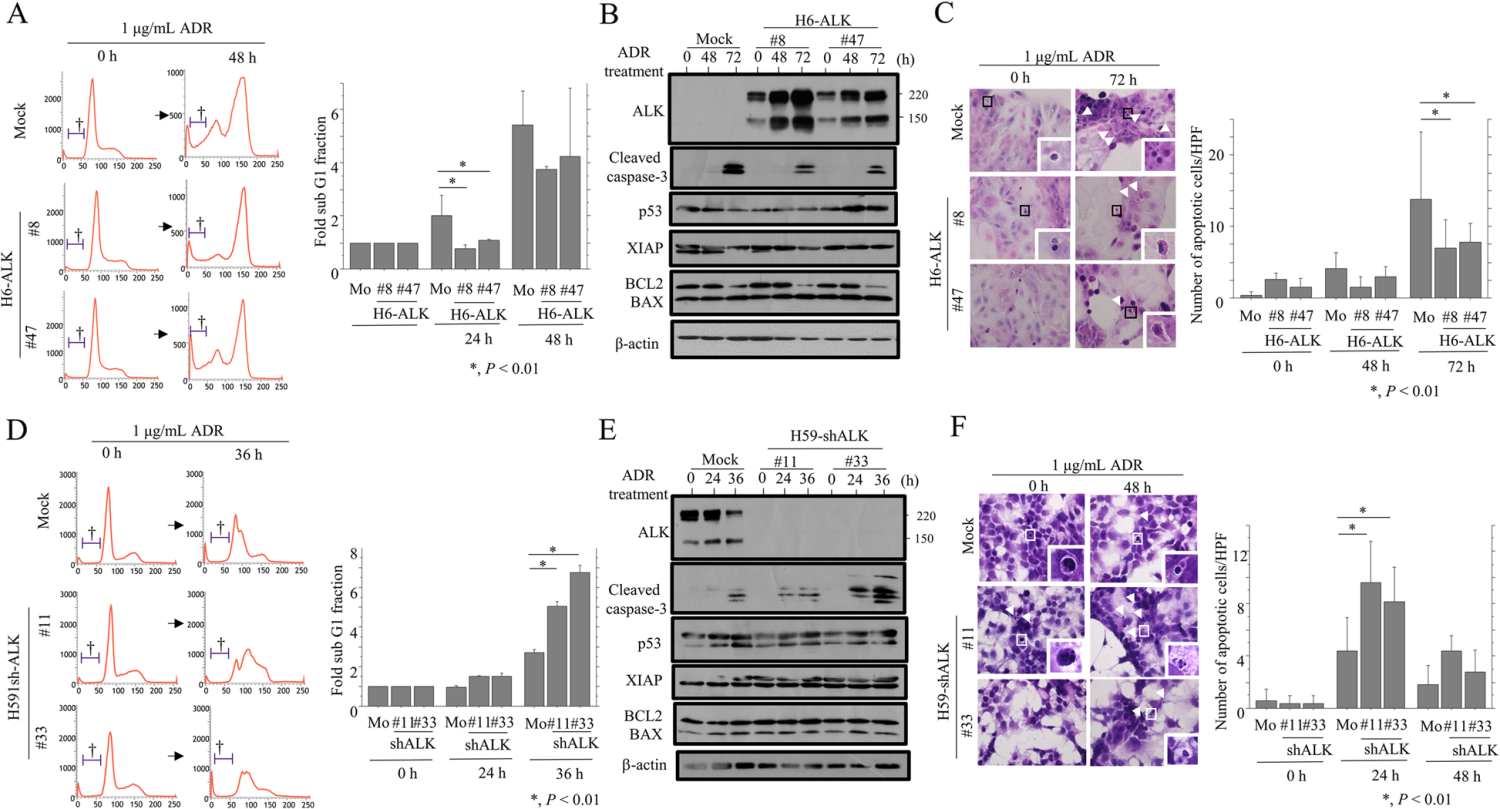
Relationship between ALK expression and apoptosis in Em Ca cells. **A**, **D** Left: Flow cytometry analysis of H6-ALK (**A**), H59-shALK (**D**), and mock cells treated with 1 μg/mL Adriamycin (ADR) for the time shown. † symbols indicate the sub-G1 fraction. Right: fold sub-G1 fractions of H6-ALK (**A**), H59-shALK (**D**), and mock cells treated with 1 μg/mL Adriamycin (ADR) for the time shown. The sub-G1 fraction values in 0 h were set as 1. The data shown are presented as means ± SDs. The experiments were performed in quintuplicate. Statistical analyses were carried out using the Mann–Whitney *U*-test. Mo, mock control. **B**, **E** Western blot analysis for the indicated proteins in total lysates from H6-ALK (**B**), H59-shALK (**E**), and mock cells treated with 1 μg/mL ADR for the time shown. **C**, **F** Left: after treatment with 1 mg/mL ADR from the time shown, H6-ALK (**C**), H59-shALK (**F**) and mock cells undergoing apoptosis are indicated by arrow-heads. Closed boxes are magnified in the insets. Original magnification, × 200 and × 400 (inset). Right: numbers of apoptotic cells are shown in means ± SDs. The experiments were performed in triplicate. Statistical analyses were carried out using the Mann–Whitney *U*-test. Mo, mock control

Compared with mock-transfected cells, ADR treatment of H6-ALK cells induced a less prominent sub-G1 FACS profile (Fig. 3A), less caspase-3 cleavage (Fig. 3B and Supplementary Figure S3A), and fewer apoptotic features (Fig. 3C). The opposite effects were observed in ADR-treated H59-shALK cells (Fig. 3D,E,F and Supplementary Figure S3B).

**Fig. 4:**
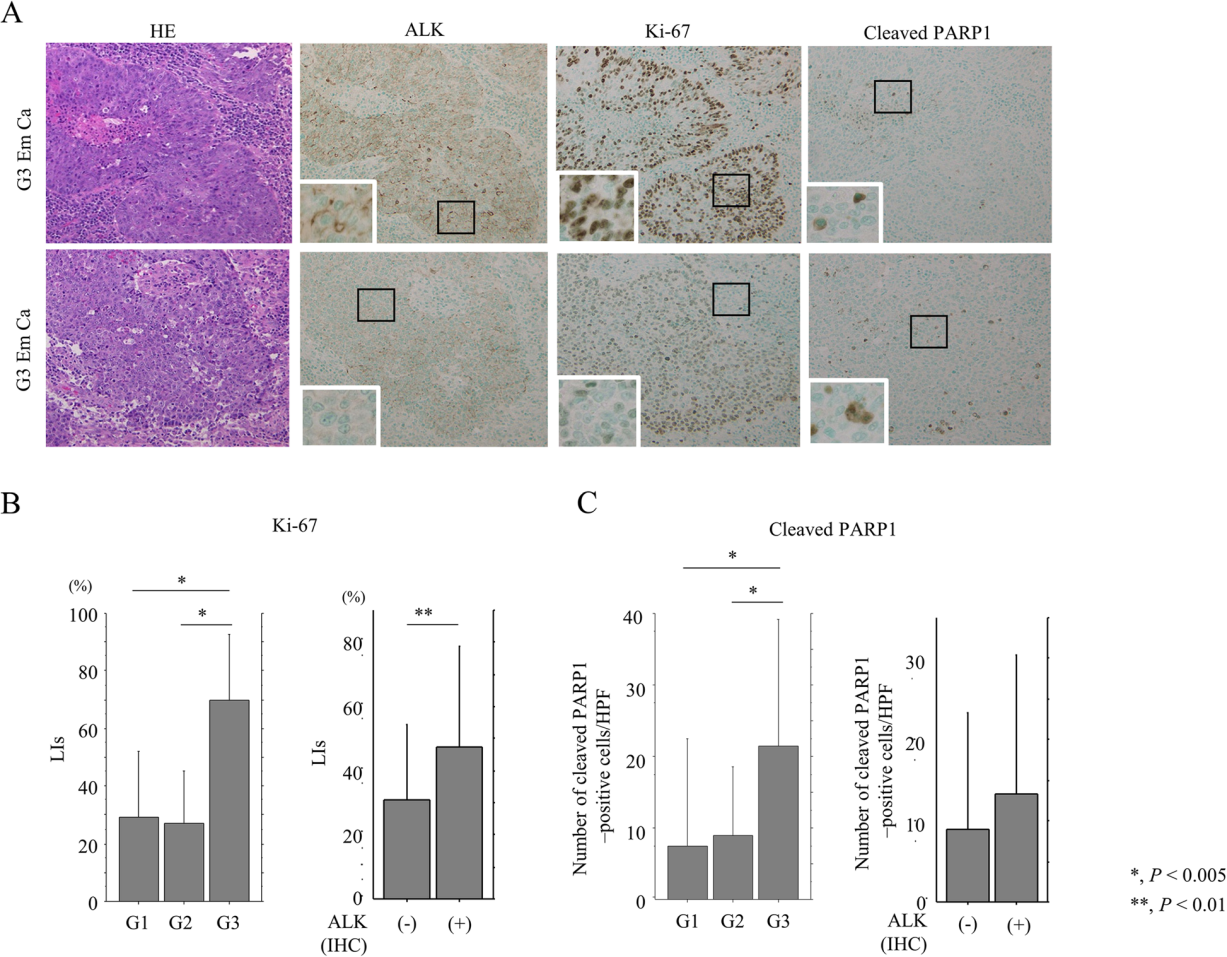
Relationship between ALK expression, cell proliferation, and apoptosis in Em Ca tissues. **A** HE and IHC staining for ALK, Ki-67, and cleaved PARP1 in G3 Em Ca. Closed boxes are magnified in the insets. Original magnification, × 100 and × 400 (inset). **B**, **C** Left: Ki-67 LIs (**B**) and number of cleaved PARP1-positive cells (**C**) between G1, G2, and G3 Em Ca. Right: Ki-67 LIs (**B**) and number of cleaved PARP1-positive cells (**C**) between ALK-positive and -negative Em Ca. The data shown are means ± SDs. Statistical analyses were carried out using the Mann–Whitney *U*-test

Figure 4A shows representative IHC images for Ki-67 and cleaved PARP1 in ALK-high and ALK-low G3 Em Ca cases. There was a positive correlation between the two markers in Em Ca (Table 1). Both Ki-67 and cleaved PARP1 immunoreactivities were significantly higher in G3 Em Ca than in G1/G2 tumors. Average Ki-67 LI values were also significantly higher in ALK-positive tumors when compared with their ALK-negative counterparts (Fig. 4B), and ALK score was also positively and significantly correlated with Ki-67 LI (Table 1). However, there were no correlations between ALK levels and cleaved PARP1 score (Fig. 3C and Table 1).

**Table 1:** Correlation between expression of ALK and other molecules in endometrial carcinoma

	ALK	CD56	SYN	p53	Me-CD133	Cyt-CD133	ALDH1	Sox2	Sox7	Ki67
	*ρ* (*P*)	*ρ* (*P*)	*ρ* (*P*)	*ρ* (*P*)	*ρ* (*P*)	*ρ* (*P*)	*ρ* (*P*)	*ρ* (*P*)	*ρ* (*P*)	*ρ* (*P*)
CD56	0.42	*	*	*	*	*	*	*	*	*
	(< 0.0001)									
SYN	0.49	0.47	*	*	*	*	*	*	*	*
	(< 0.0001)	(< 0.0001)								
p53	0.45	0.17	0.34	*	*	*	*	*	*	*
	(< 0.0001)	-0.06	-0.0002							
Me-CD133	0.22	0.06	0.05	0.1	*	*	*	*	*	*
	0.01	-0.4	-0.5	-0.2						
Cyt-CD133	0.59	0.41	0.49	0.35	0.47	*	*	*	*	*
	(< 0.0001)	(< 0.0001)	(< 0.0001)	(0.0001)	(< 0.0001)					
ALDH1	0.4	0.26	0.31	0.14	0.08	0.4	*	*	*	*
	(< 0.0001)	-0.004	-0.0004	(0.1)	(0.4)	(< 0.0001)				
Sox2	0.6	0.42	0.41	0.41	0.16	0.6	0.38	*	*	*
	(< 0.0001)	(< 0.0001)	(< 0.0001)	(< 0.0001)	(0.08)	(< 0.0001)	(< 0.0001)			
Sox7	0.54	0.31	0.37	0.38	0.17	0.17	0.34	0.49	*	*
	(< 0.0001)	-0.001	(< 0.0001)	(< 0.0001)	(0.07)	(0.07)	(0.0002)	(< 0.0001)		
Ki-67	0.43	0.32	0.41	0.46	-0.17	-0.17	0.2	0.55	0.31	*
	(< 0.0001)	-0.0006	(< 0.0001)	(< 0.0001)	(0.06)	(0.06)	(0.02)	(< 0.0001)	(0.0007)	
Cleaved	0.33	0.31	0.47	0.44	-0.08	-0.08	0.16	0.5	0.26	0.73
PARP1	(0.0001)	(0.001)	(< 0.0001)	(< 0.0001)	(0.4)	(0.4)	(0.07)	(< 0.0001)	(0.005)	(< 0.0001)

*Me* membrane, *Cyt* cytoplasmic

Statistical analyses were performed using the Spearman's correlation coefficient

These findings suggest that ALK overexpression increases proliferation in Em Ca cell lines and clinical samples, and reduces susceptibility to apoptosis in the former but not the latter.

### ALK overexpression enhances CSC features and migration in Em Ca cells

**Fig. 5:**
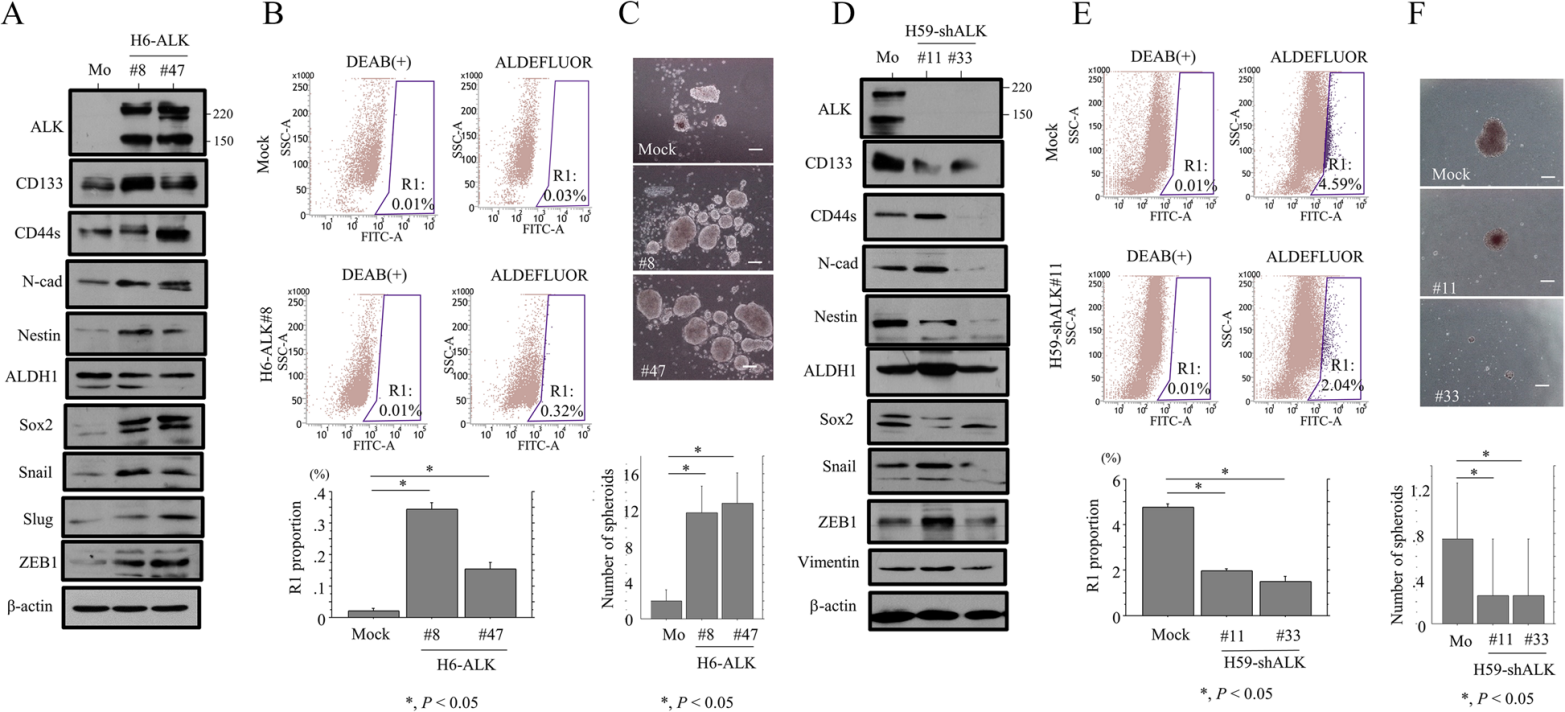
Changes in CSC properties in Em Ca cells. **A**, **D** Western blot analysis for the indicated proteins in total lysates from H6-ALK (**A**), H59-shALK (**D**), and mock cells (Mo). **B**, **E** Upper and middle: Aldefluor analysis of H6-ALK (**B**), H59-shALK (**E**), and mock cells. Cells with no ALDH1 activity are located in the area to the far left of each plot, and the positive cells are within the black gate (R1). The percentage of live single cell populations contained in each gate is shown. DEAB, dimethylaminobenzaldehyde. Lower: percentage of R1 proportion in H6-ALK (**B**), H59-shALK (**E**), and mock cells. The experiments were performed in triplicate. The data shown are means ± SDs. Statistical analyses were carried out using the Mann–Whitney *U*-test. **C**, **F** Upper: phase-contrast photograms of spheroids derived from H6-ALK (**C**), H59-shALK (**F**), and mock cells following 2 weeks of growth. Scale bars = 50 mm. Lower: numbers of spheroids are presented as means ± SDs. The experiments were performed in triplicate. Statistical analyses were carried out using the Mann–Whitney *U*-test. Mo, mock controls; N-cad, N-cadherin

ALK induces CSC features through activation of the epithelial-mesenchymal transition (EMT) in ovarian high-grade serous carcinomas and uterine carcinosarcoma [18, 19]. We therefore examined whether the association between ALK expression and CSC properties was also feature of Em Ca. Indeed, H6-ALK cells exhibited increased expression of several CSC and EMT markers including CD133, CD44s, N-cadherin, Nestin, Sox2, Snail, Slug, and ZEB1 when compared to mock cells (Fig. 5A and Supplementary Figure S4A). The Aldefluor assay also revealed a significant ALDH1^high^ population in the H6-ALK cells (Fig. 5B), in line with a significant increase in the number of well-defined, round spheroids that were over 50 mm in diameter (Fig. 5C). In contrast, levels of these markers were significantly lower in H59-shALK cells (Fig. 5D, E, F and Supplementary Figure S4B).

**Fig. 6:**
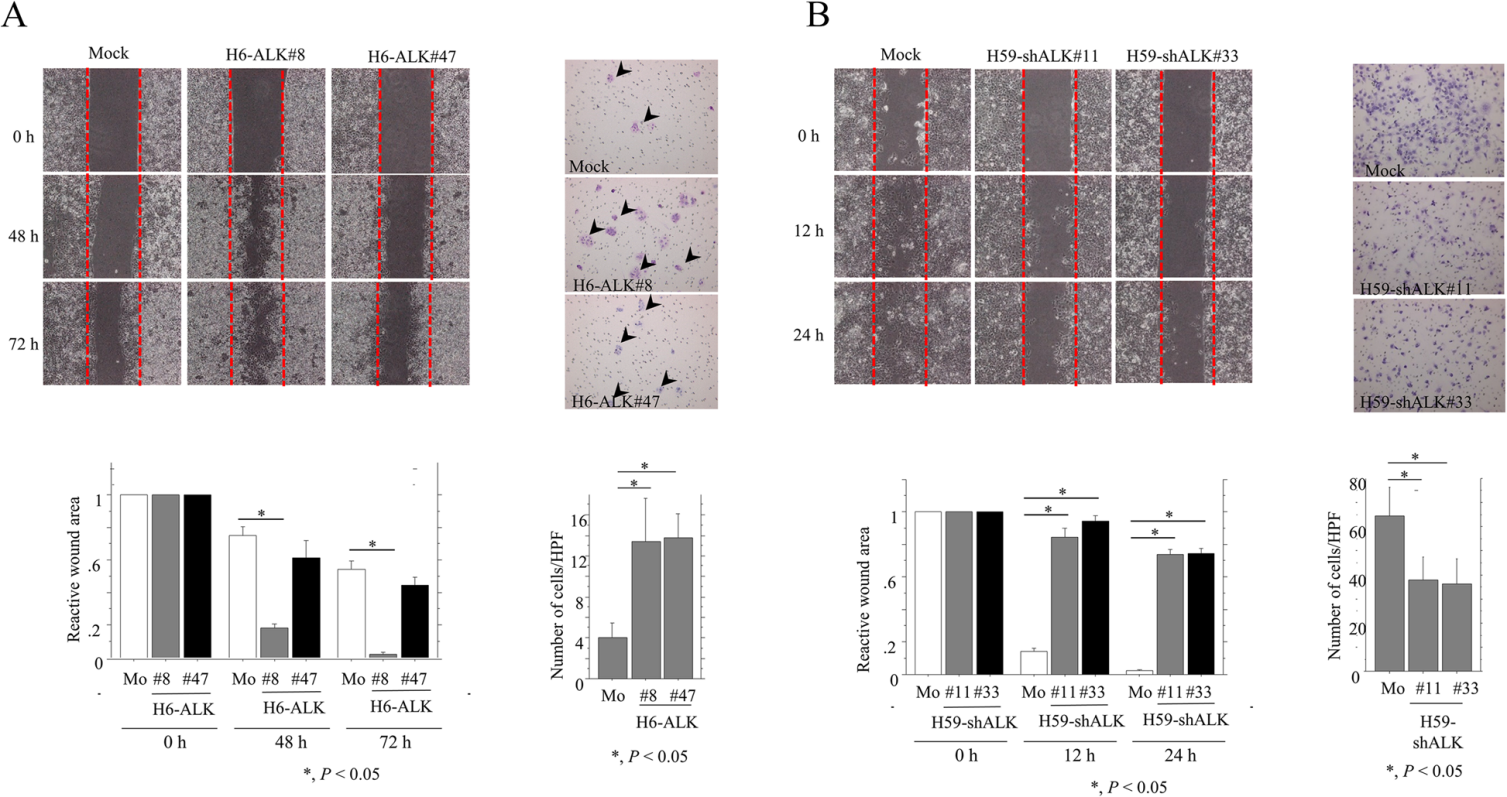
Change in cell migration potential of Em Ca cells. **A**, **B** Left upper: wound healing assay with H6-ALK (**A**), H59-shALK (**B**), and mock cells. A scratch was made in the middle of a layer of confluent cells, and phase contrast images were taken over the indicated time period. The red dotted lines indicate the borders between confluent cell layers and wound areas. Left lower: the values of wound areas were calculated using NIH ImageJ software with those at 0 h set as 1. The fold wound areas are presented as means ± SDs. Mo, mock controls. The experiment was performed in triplicate. Statistical analyses were carried out using the Mann–Whitney *U*-test. Right upper: H6-ALK, H59-shALK and mock cells were seeded in a 24-well transwell plates and incubated for 24 h in medium without serum. Cells were stained with HE and counted using a light microscope. Right lower: the numbers of migrated cells are presented as means ± SDs. Mo, mock controls. The experiment was performed in triplicate. Statistical analyses were carried out using the Mann–Whitney *U*-test

We then examined whether ALK expression modulates cell motility using the wound healing assay. H6-ALK cells refilled wounded empty spaces more rapidly, and exhibited significantly increased migration capacity (Fig. 6A). Conversely, H59-shALK cells were less able to refill the empty wound spaces, and had lower migration rates than mock cells (Fig. 6B).

**Fig. 7:**
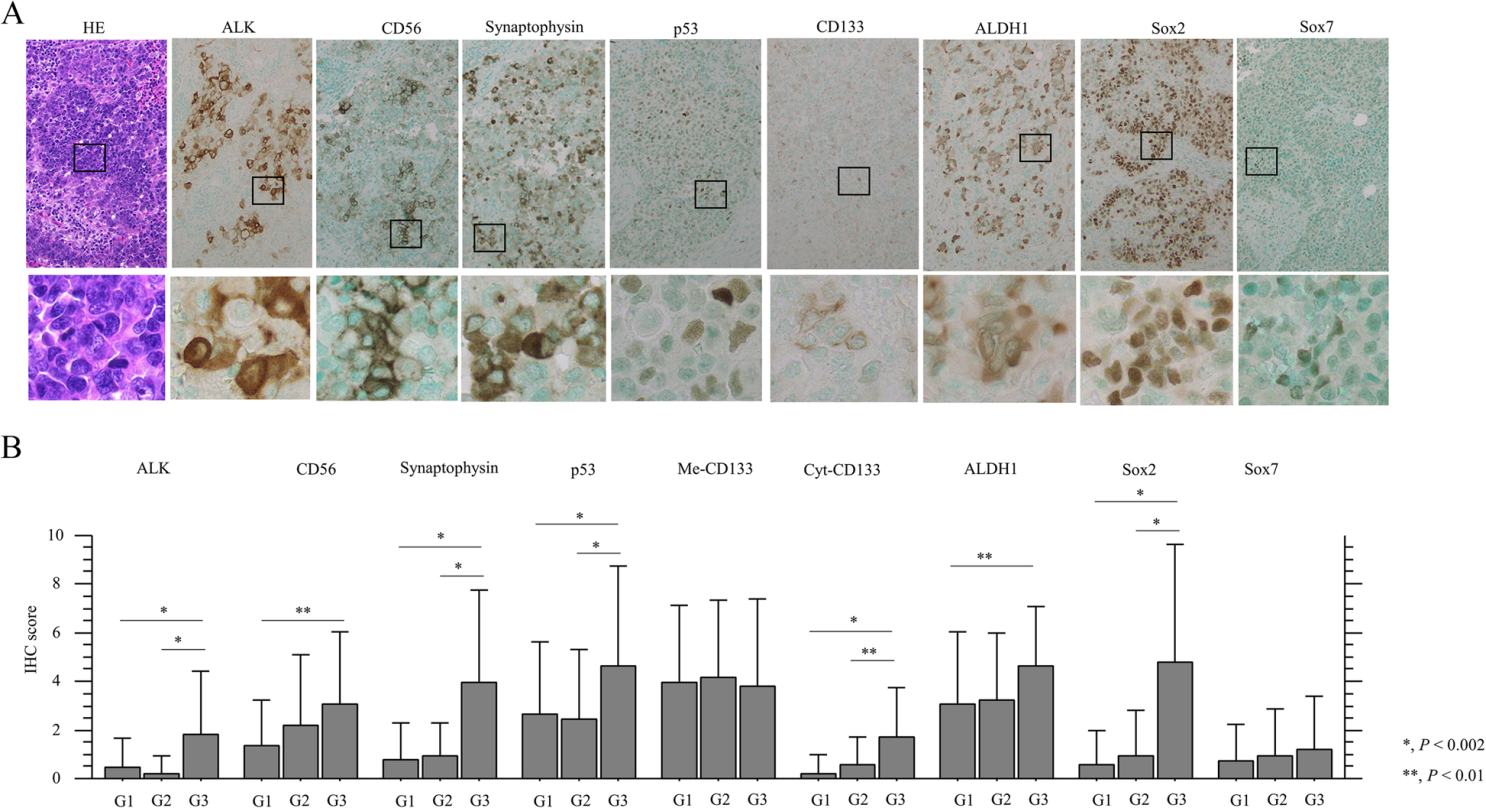
IHC in serial sections of Em Ca tissues. **A** Staining by HE and IHC for the indicated proteins in G3 Em Ca. Closed boxes are magnified in the insets. Original magnification, × 100 and × 400 (inset). **B** IHC scores for the indicated proteins in Em Ca. The data shown are means ± SDs. Statistical analyses were carried out using the Mann–Whitney *U*-test

Since ALK overexpression is associated with neuroendocrine (NE) differentiation and *TP53* gene status in ovarian high-grade serous carcinomas and uterine carcinosarcoma [18, 19], we investigated NE markers and p53 staining in Em Ca. Distinct cytoplasmic and/or membranous immunostaining for CD56, Syn, CD133, and ALDH1, as well as nuclear immunoreactivity for p53, Sox2, and Sox7 were observed in Em Ca cells (Fig. 7A). Average IHC scores for CD56, Syn, p53, cytoplasmic (Cyt)-CD133, ALDH1, and Sox2, as well as ALK, were significantly higher in G3 Em Cas as compared to those of G1 or G2 tumors, but this differential pattern was not evident for membranous CD133 or Sox7 (Fig. 7B). In addition, ALK score was positively correlated with CD56, Syn, Cyt-CD133, ALDH1, Sox2, Sox7, and p53 scores. Both Sox2 and Cyt-CD133 scores were also positively associated with a subset of NE and CSC markers (Table 1).

These findings suggest that ALK overexpression enhances CSC properties, migration capability, and NE features in Em Ca cells.

### TCGA data analysis for correlation between ALK status and survival in Em Ca

Kaplan–Meier curves showed that Em Ca patients with high ALK mRNA expression and wild-type *ALK* gene status had poorer OS and PFS when compared to patients with low ALK mRNA expression and mutant *ALK* (Supplementary Figure S5).

## Discussion

The present study clearly provides evidence of a positive correlation between ALK mRNA and protein expression in Em Ca tissues, in contrast to a lack of ALK expression in any of the normal endometrial components, suggesting that ALK overexpression may be a tumor-specific feature of Em Ca. In general, mutated or fused ALK kinases are autophosphorylated and display increased kinase activity when compared to the wild-type kinase [17, 29]. *ALK* mutations are frequently found in exons 20 and 23–25, which encode portions of the juxtamembrane domain and tyrosine kinase domain, respectively [28]. However, we detected neither ALK chromosomal rearrangements nor mutations in the clinical samples and cell lines used in our study.

We previously showed that full length ALK overexpression was under the transcriptional control of SoxB1 (Sox2 and Sox3) and SoxF (Sox7 and Sox17) in ovarian high-grade serous carcinoma [18], suggesting the existence of a positive feedback loop between ALK and Sox factors. We also found that *ALK* promoter activity was suppressed by wild-type p53, but not mutant p53 [18]. A high p53 score (60—100% immunopositive cells) is sufficient to identify a p53 mutation in 94% of cases [30]. Given our present finding that ALK expression was positively correlated with Sox2, Sox7, and p53 scores in Em Ca tissues, we suggest that transcriptional upregulation of ALK may also occur in Em Ca via transcriptional activators including Sox factors and p53.

We also found that ALK overexpression was significantly higher in G3 Em Ca when compared to G1/G2 tumors, and that ALK was predominantly cytoplasmic in G3 tumors, and membrane-localized in G1/G2 tumors. Moreover, ALK expression was associated with increased proliferation, suggesting that cytoplasmic ALK may contribute to the aggressive behavior of Em Ca, probably through modulation of proliferative capability. This conclusion is supported by previously published reports. First, high cytoplasmic ALK expression is significantly associated with several unfavorable clinicopathological factors and poor prognosis in ovarian high-grade serous carcinoma [18]. Second, cytoplasmic localization of the ALK tyrosine kinase domain promotes proliferation, whereas membrane attachment is critical for initiation of neurite outgrowth and proliferation arrest in PC12 cells [31]. Finally, universal cytoplasmic ALK expression is also widely observed in neuroblastomas, suggesting that some transcriptional or posttranslational regulation of the ALK might exist in these tumor cells [32].

In this study, stable ALK overexpression in Em Ca cell lines significantly increased proliferation and reduced susceptibility to apoptosis, and these effects were abrogated in ALK-knockdown cells. This is probably mediated via activation of ALK-related signaling pathways including phosphatidylinositol 3-kinase-AKT, Janus kinase-STAT, and mitogen-activated protein kinase pathways, which contribute to regulation of cell cycle progression and apoptotic features [33]. However, we failed to demonstrate any association between ALK overexpression and apoptotic features determined by cleaved PARP1 immunohistochemistry in clinical Em Ca tissues. One possible explanation for these discordant results is that ALK overexpression may be indirectly associated with modulation of apoptosis in Em Ca cells. In addition, we found that ALK was upregulated in response to either serum stimulation or ADR treatment in H6-ALK cells. We therefore speculate that ALK is post-translationally stabilized in response to proliferative and apoptotic stimuli. Further studies regarding this final point are clearly warranted.

An important finding of this study is that ALK overexpression enhanced CSC properties; this is consistent with the upregulation of several CSC markers, including Cyt-CD133, ALDH1, and Sox2 in Em Ca tissues. Moreover, ALK overexpression significantly enhanced migration capability, whereas ALK knockdown abrogates this effect. In glioblastoma, CSCs are more invasive than non-CSCs [34]. Taking these results together, we infer that the increased migration capability of CSC-like cells may contribute to the aggressive features of ALK-overexpressing Em Ca cells.

Finally, overexpression of ALK, as well as Sox2, was also clearly associated with expression of NE markers in G3 Em Ca cells. Similar findings were also observed in ovarian high-grade serous carcinoma [18]. The prognosis of NE carcinoma is poor, as more than 80% of patients present with metastatic disease at diagnosis, and there are few effective therapies for this cancer type [35]. Given that Sox2 blocks the differentiation of the neural progenitor population [36], we suggest that the combined activities of ALK and Sox2 induce an NE-like phenotype in Em Ca cells, and that this contributes to the poor OS and PFS in Em Ca. Our hypothesis was supported by analysis of TCGA data, which showed that high ALK mRNA expression and wild-type *ALK* status were associated with poorer prognosis in Em Ca cases.

## Conclusion

**Fig. 8:**
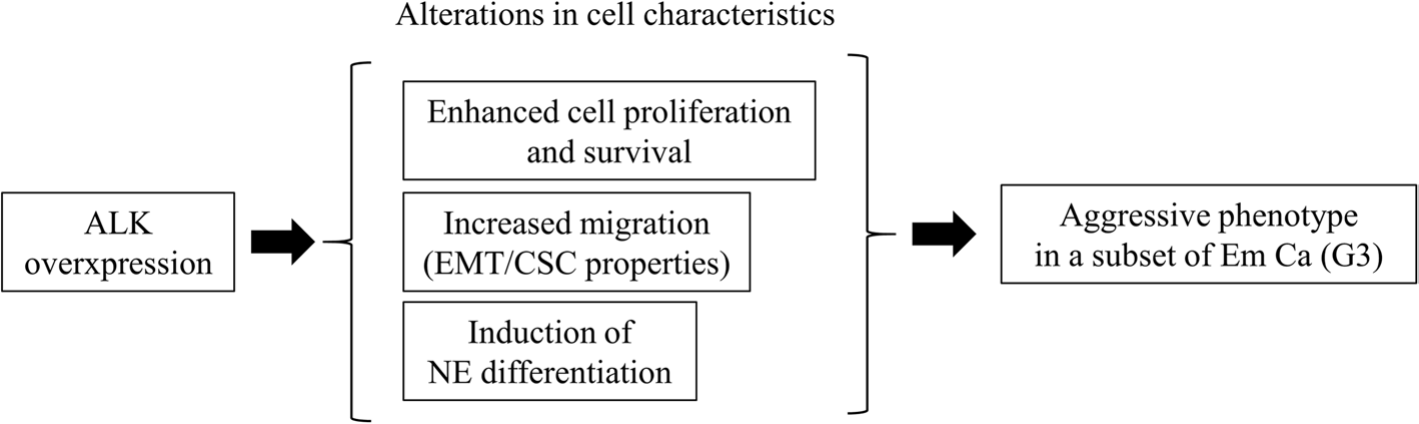
Schematic representation of the functional roles of ALK in an aggressive phenotype of Em Ca

Our overall conceptual framework for the role of ALK in aggressive Em Ca is summarized in Fig. 8. Full-length ALK overexpression occurs in a subset of Em Ca, particularly in G3 tumors, and contributes to establishment and maintenance of aggressive phenotypic characteristics through modulation of several biological processes.

### Supplementary Information

**Additional file 1: Supplementary Figure S1.** ALK expression in Em Ca cells. (A) ALK mRNA expression in 11 Em Ca cell lines. Note the strong mRNA signals in Hec59 and Hec251 cells and the weak signals in Ishikawa and Hec88 cells. (B) CCLE data analysis for ALK mRNA expression in 28 Em Ca cell lines, demonstrating the high ALK mRNA expression in Hec59 cells and the low level in Hec251 cells. (C) Western blot analysis for the indicated proteins in total lysates from Hec59 and Hec251 cells. Note the full length ALK protein expression (220 kDa) in Hec59 but not Hec251 cells. D) Mutation analysis of exons 20, 23, 24, and 25 of the *ALK* gene in Hec59 cells, demonstrating a lack of mutations in the four exons. (E) H6-ALK#47 cells are stained with anti-ALK antibody. Note the cytoplasmic ALK staining (indicated by arrows). Original magnification, x200.

**Additional file 2: Supplementary Figure S2.** Original images of western blot analysis for the indicated proteins in total lysates from H6-ALK (A), H59-shALK (B), and mock cells.

**Additional file 3: Supplementary Figure S3.** Original images of western blot analysis for the indicated proteins in total lysates from H6-ALK (A), H59-shALK (B), and mock cells.

**Additional file 4: Supplementary Figure S4.** Original images of western blot analysis for the indicated proteins in total lysates from H6-ALK (A), H59-shALK (B), and mock cells. The predictive sizes are indicated by arrows. N-cad, N-cadherin.

**Additional file 5: Supplementary Figure S5. **TCGA data analysis for associations between ALK status and prognosis in Em Ca. OS (left) and PFS (right) relative to ALK mRNA (A) and the gene mutation status (B). n, number of cases. Statistical analyses were performed using the log rank test.

**Additional file 6: Supplementary Table S1.** Relationship between ALK expression and clinicopathological factors in endometrial carcinoma.

**Additional file 7: Supplementary Table S2.** ALK mRNA expression and mutations in endometrial carcinoma cell lines.

## Data Availability

The data sets generated during and/or analyzed during the current study are available from the corresponding author on reasonable request.

## Abbreviations

ALK: Anaplastic lymphoma kinase
Em Ca: Endometrial carcinoma
CSC: Cancer stem cell
EMT: Epithelial-mesenchymal transition
IHC: Immunohistochemistry
NE: Neuroendocrine
