# Supplementary figures and images for: Anaplastic lymphoma kinase overexpression enhances aggressive phenotypic characteristics of endometrial carcinoma
Source: BMC Cancer. 2023 Aug 17;23:765. doi: 10.1186/s12885-023-11144-2 (PMC10436652; doi:10.1186/s12885-023-11144-2)

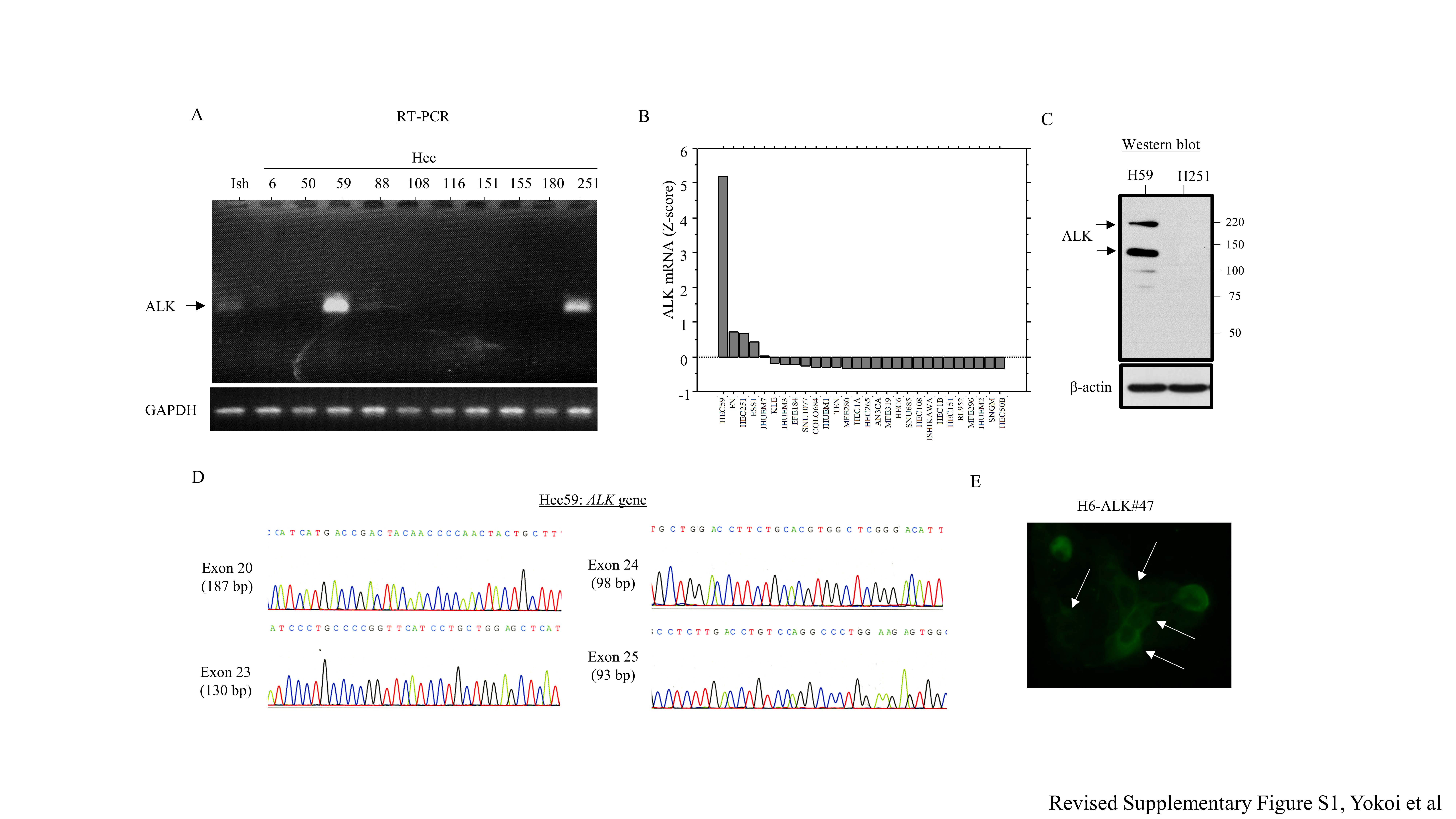

Supplement: Supplementary file 1 — Additional file 1: Supplementary Figure S1. ALK expression in Em Ca cells. (A) ALK mRNA expression in 11 Em Ca cell lines. Note the strong mRNA signals in Hec59 and Hec251 cells and the weak signals in Ishikawa and Hec88 cells. (B) CCLE data analysis for ALK mRNA expression in 28 Em Ca cell lines, demonstrating the high ALK mRNA expression in Hec59 cells and the low level in Hec251 cells. (C) Western blot analysis for the indicated proteins in total lysates from Hec59 and Hec251 cells. Note the full length ALK protein expression (220 kDa) in Hec59 but not Hec251 cells. D) Mutation analysis of exons 20, 23, 24, and 25 of the ALK gene in Hec59 cells, demonstrating a lack of mutations in the four exons. (E) H6-ALK#47 cells are stained with anti-ALK antibody. Note the cytoplasmic ALK staining (indicated by arrows). Original magnification, x200. [file 12885_2023_11144_MOESM1_ESM.tif]

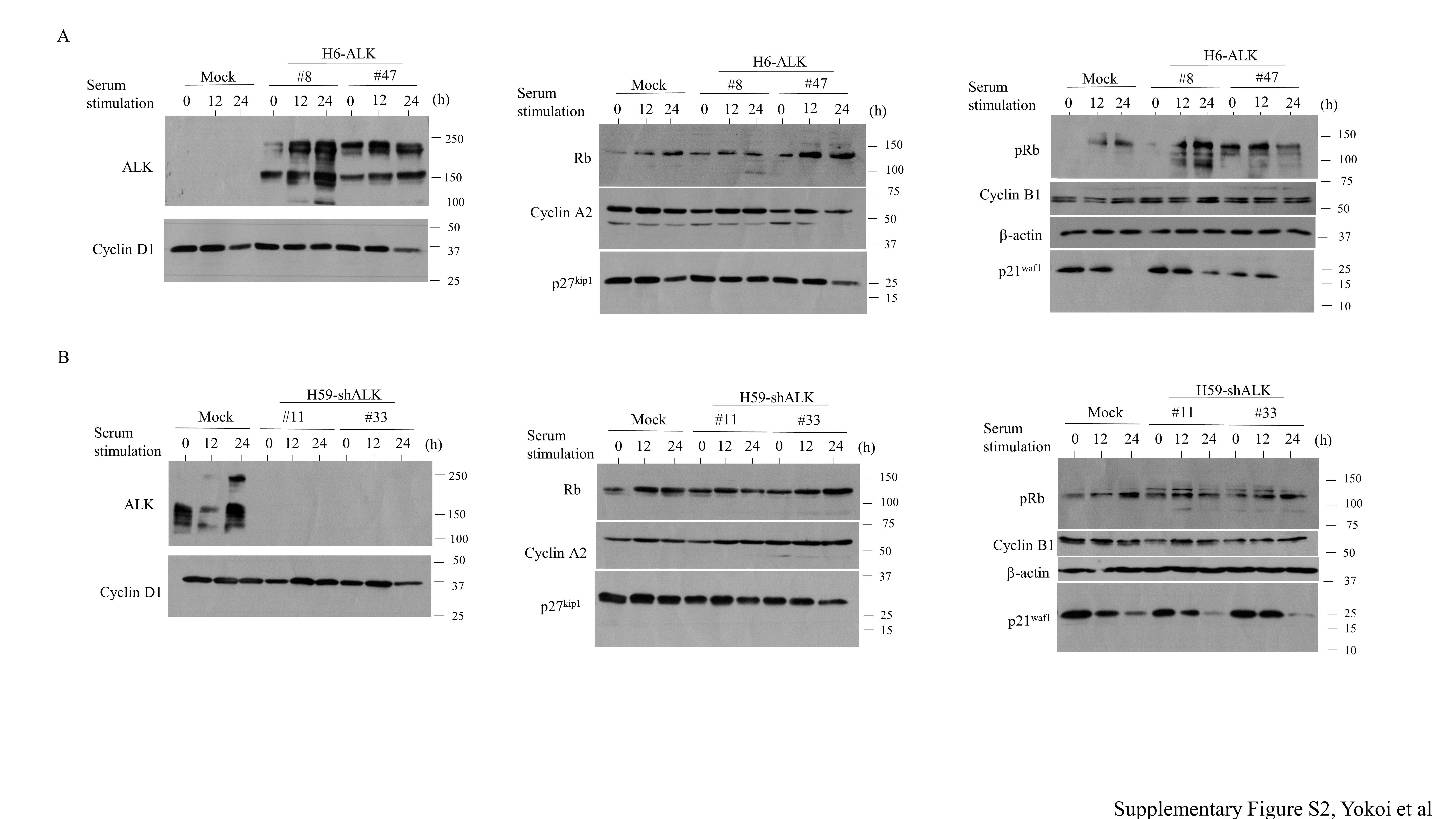

Supplement: Supplementary file 2 — Additional file 2: Supplementary Figure S2. Original images of western blot analysis for the indicated proteins in total lysates from H6-ALK (A), H59-shALK (B), and mock cells. [file 12885_2023_11144_MOESM2_ESM.tif]

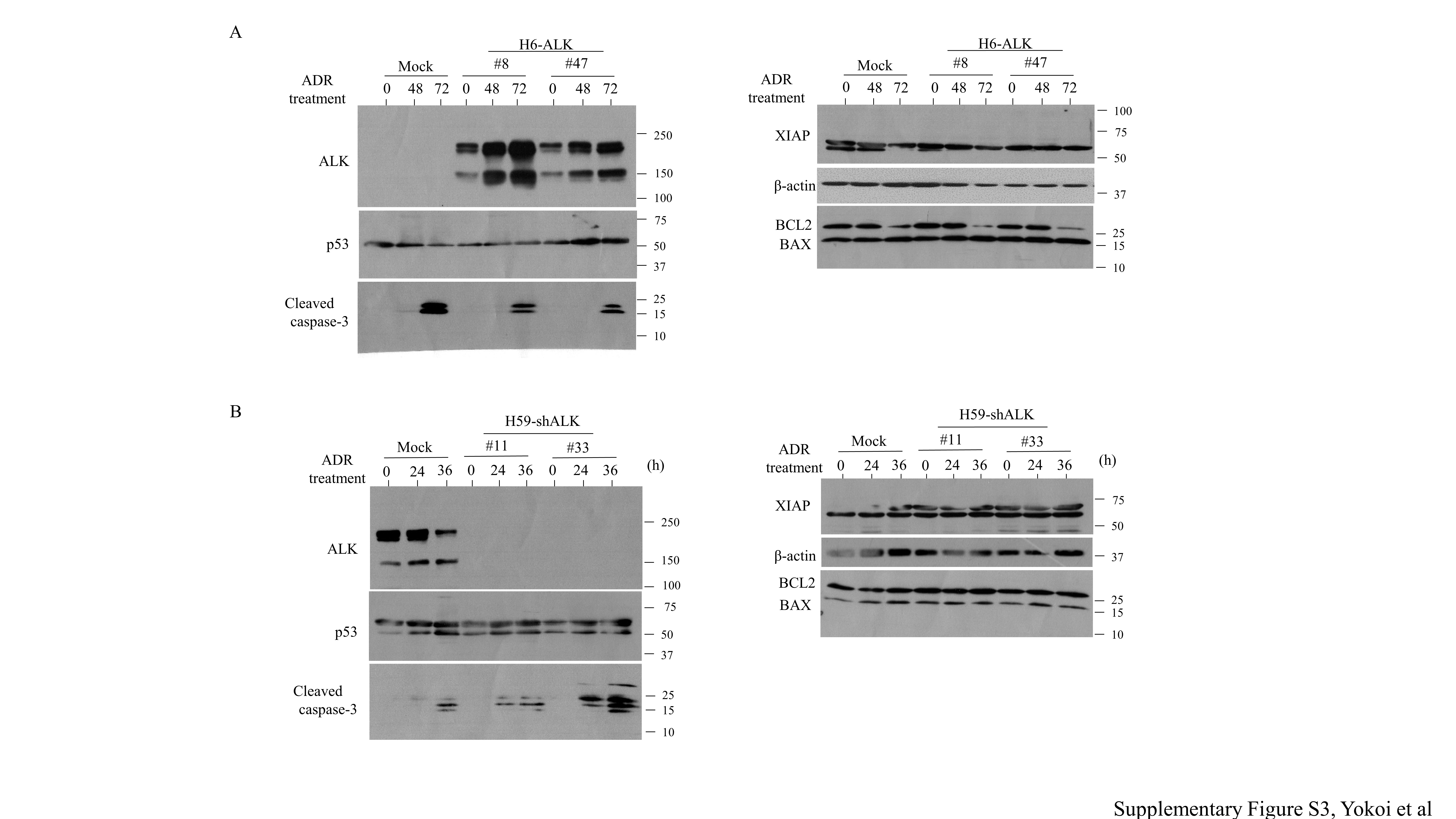

Supplement: Supplementary file 3 — Additional file 3: Supplementary Figure S3. Original images of western blot analysis for the indicated proteins in total lysates from H6-ALK (A), H59-shALK (B), and mock cells. [file 12885_2023_11144_MOESM3_ESM.tif]

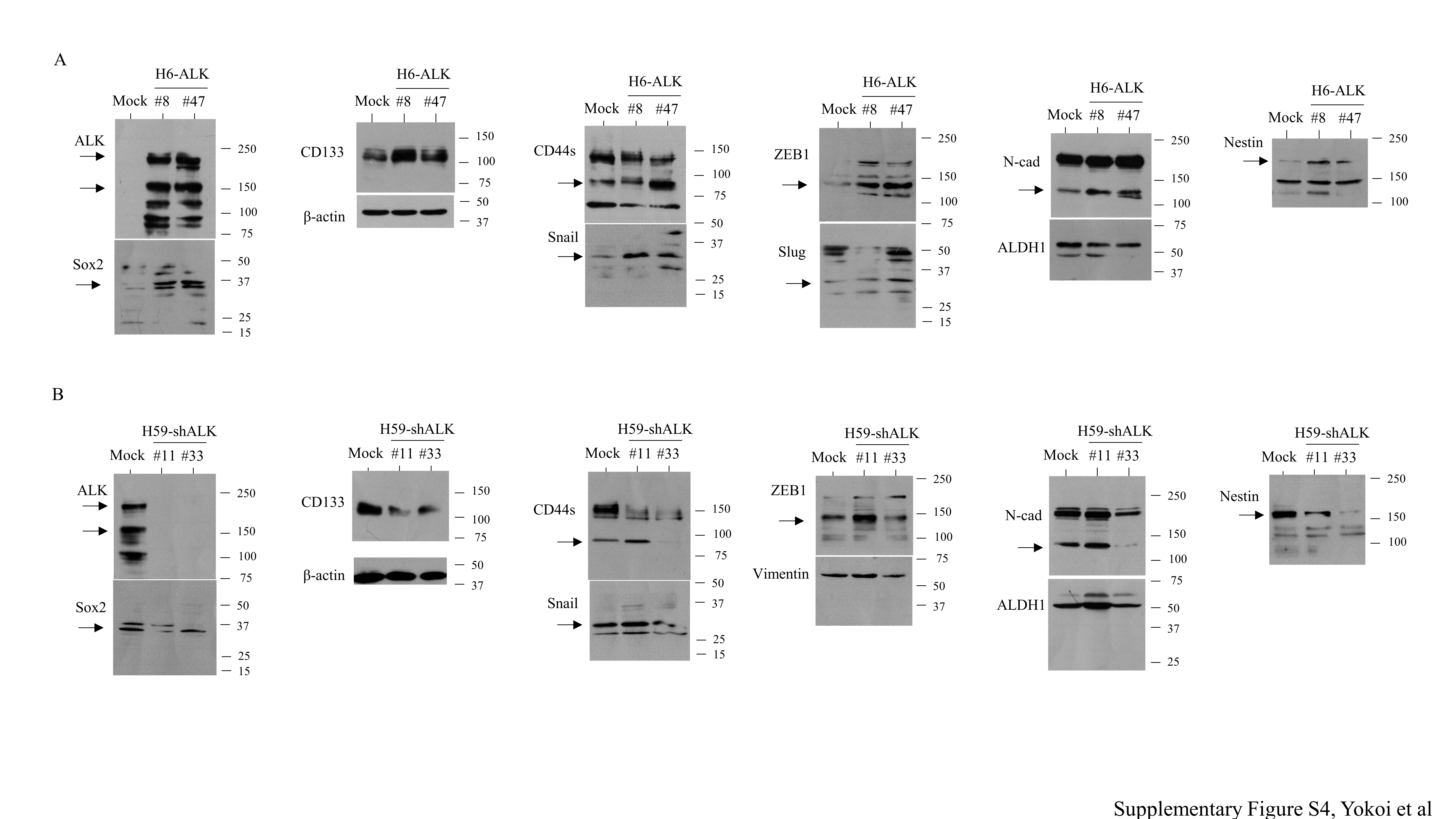

Supplement: Supplementary file 4 — Additional file 4: Supplementary Figure S4. Original images of western blot analysis for the indicated proteins in total lysates from H6-ALK (A), H59-shALK (B), and mock cells. The predictive sizes are indicated by arrows. N-cad, N-cadherin. [file 12885_2023_11144_MOESM4_ESM.tif]

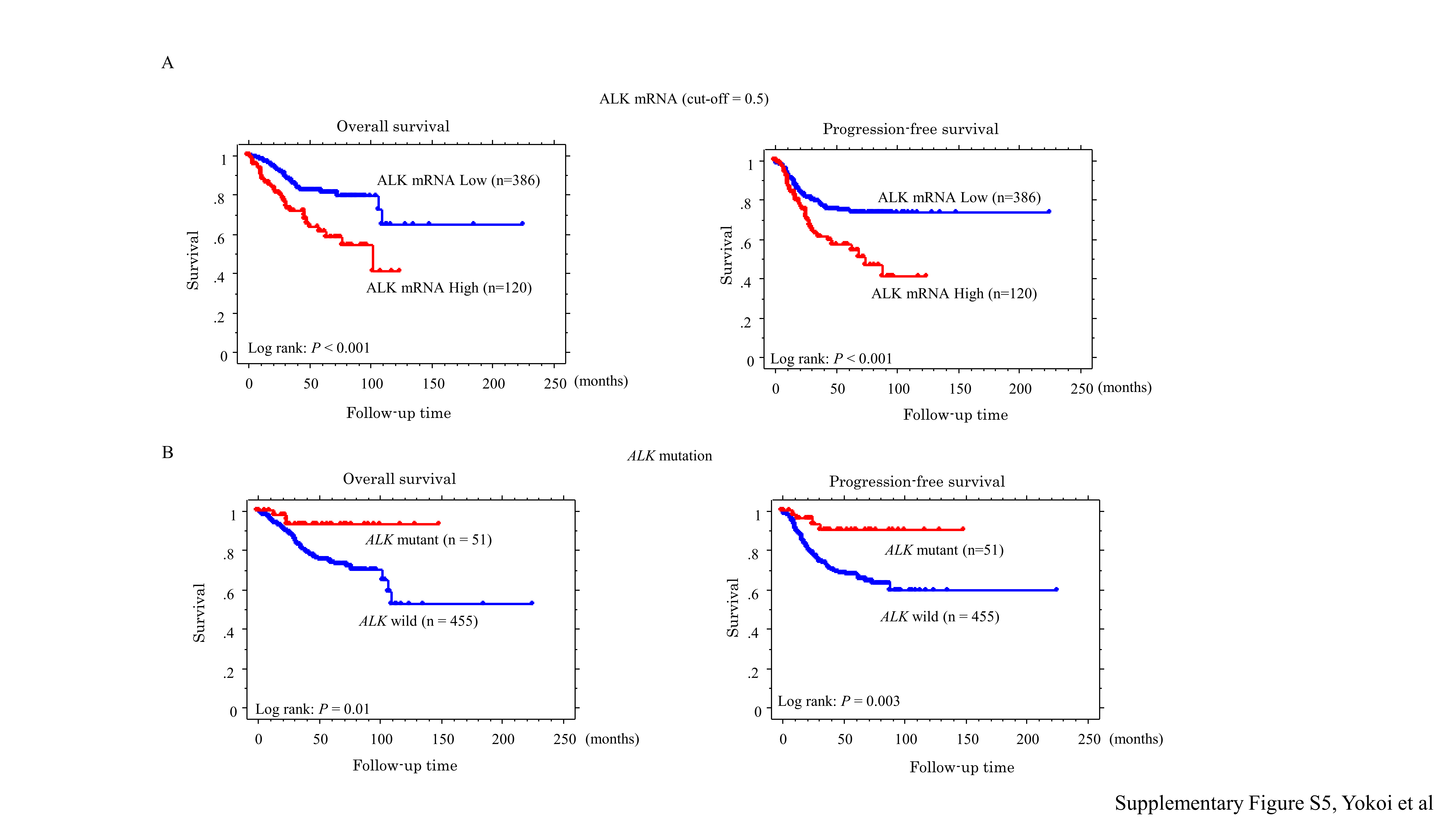

Supplement: Supplementary file 5 — Additional file 5: Supplementary Figure S5. TCGA data analysis for associations between ALK status and prognosis in Em Ca. OS (left) and PFS (right) relative to ALK mRNA (A) and the gene mutation status (B). n, number of cases. Statistical analyses were performed using the log rank test. [file 12885_2023_11144_MOESM5_ESM.tif]
